# Hard to jump: host shifts appear unlikely in a T4-like phage evolved in the lab

**DOI:** 10.3389/fcimb.2025.1597805

**Published:** 2025-11-25

**Authors:** Yu Ning, Enrique González-Tortuero, Jeroen Wagemans, Flor I. Arias-Sánchez

**Affiliations:** 1 BIH Center for Regenerative Therapies (BCRT), Charité - Universitätsmedizin Berlin, Berlin, Germany; 2 School of Science, Engineering and Environment, University of Salford, Salford, United Kingdom; 3 Department of Biosystems, Katholieke Universiteit (KU) Leuven Kulak, Kortrijk, Belgium; 4 Institute of Integrative Biology, ETH Zürich, Zürich, Switzerland

**Keywords:** phage therapy, host shifts, virulence, infection, phage therapeutics

## Abstract

**Introduction:**

Bacteriophage therapy is emerging as a promising alternative to antibiotics, particularly in the face of rising antimicrobial resistance. However, concerns remain regarding host shifts, where therapeutic phages could evolve to infect and harm beneficial commensal bacteria. Understanding how frequently host shifts occur and what evolutionary constraints shape them is critical to assessing the safety of phage therapy.

**Methods:**

We investigated the evolutionary potential for host shifts using Escherichia coli-infecting phage BW-1. Experimental evolution was conducted under controlled conditions that favored adaptation, using both non-permissive (unable to infect) and semi-permissive (low infectivity) bacterial strains. Virulence was assayed across hosts, and whole-genome sequencing was used to identify mutations associated with adaptation.

**Results:**

Host shifts were found to be rare, with no significant increases in virulence observed in non-permissive hosts. In contrast, adaptation occurred in semi-permissive hosts and was linked to trade-offs, where increased virulence in one host reduced infectivity in others. Whole-genome sequencing revealed a single convergent regulatory SNP across all phages adapted to the semi-permissive host, indicating constrained evolutionary pathways during host adaptation.

**Discussion:**

Our findings suggest that phages exhibit high host specificity, which limits the risk of host shifts to commensal bacteria. Although adaptation to semi-permissive hosts is possible, it is constrained and associated with fitness trade-offs across host ranges. These results indicate that therapeutic phages are unlikely to negatively impact intestinal microbiota, supporting their potential as safe and effective alternatives to antibiotics.

## Introduction

When they shift to a new host, parasites can overcome their overall specificity and gain access to new environments. Although host shifts are beneficial to the parasite, they can be highly detrimental to the novel host, as demonstrated by the number of human pandemics (like HIV, malaria and influenza) that are caused by newly acquired parasites (Liu et al., 2010; Mollentze and Streicker, 2020; Sharp and Hahn, 2010; Webby and Webster, 2001). Despite our knowledge of host shifts as natural events that tend to occur among closely related species (Lee, 2024; Longdon et al., 2014; Seal et al., 2021; Woolhouse et al., 2005), understanding what drives and constrains host shifts remains elusive. We lack information about how frequently they happen and whether they tend to be associated with increased virulence in the new or old hosts.

A better understanding of the conditions that facilitate or constrain viral host shifts and high levels of virulence might also be crucial for the success of new treatments like phage therapy. If host shifts are facilitated among closely related hosts, using phages to treat infections in complex communities such as the gut might result in undesirable host shifts to commensal bacteria. Although patterns of phage-bacterial infection in complex communities such as those in the gastrointestinal tract are still poorly understood (Mirzaei and Maurice, 2017; Shkoporov and Hill, 2019), many factors predicted to be essential for host shifts are inherent to the host. These include CRISPR/Cas systems (Angermeyer et al., 2021; Sorek et al., 2008), the presence of particular proteins like phage receptors (Bertozzi Silva et al., 2016; Kim et al., 2023), horizontally transferred elements like plasmids (Jalasvuori et al., 2011; Shan et al., 2023), or just general phenotypic similarity to existing hosts, which would be reflected in phylogenetic distance effect (Longdon et al., 2011; Walsh et al., 2023).

Here, we looked at host shifts using a microbial system comprised of a lytic bacteriophage (virus infecting bacteria) and 8 strains of *Escherichia coli*. As a species, *E. coli* is a highly versatile continuum that ranges from commensal strains living in the gut to pathogenic strains causing urinary tract infections or severe gut infections (Kaper et al., 2004; Siniagina et al., 2021; Tenaillon et al., 2010). Phages infecting *E. coli* are specific for particular strains (Allen et al., 2017; Li et al., 2022; Michel et al., 2010). Past work shows that phages infecting *E. coli* and other bacteria can rapidly adapt to increase their infectivity against bacterial hosts and sometimes adapt to new host strains (Benmayor et al., 2009; Bohannan and Lenski, 2000; Koskella et al., 2022), but the outcomes can be highly variable. Despite this variability, few studies have addressed host shifts systematically in a controlled evolutionary framework. Our study is one of the first to experimentally approach this question using a phage with known host range variability and a rationally chosen set of diverse *E. coli* strains. We, therefore, hypothesized that a phage would be able to adapt to some novel hosts from a collection of natural and clinical isolates. We made the additional prediction that adaptation to new hosts would be most likely for hosts more genetically similar to the set of known host genotypes that the phage could already infect (native host range). To test this, we evolved a phage in the presence of various novel hosts that the phage could not infect (non-permissive host). We controlled the experimental conditions to favor host shifts. BW1 was selected as the focal phage because it exhibited diversity in its infectivity across strains, suggesting evolutionary potential in its host range. The *E. coli* strains were drawn from the well-characterized ECOR collection and were selected to represent different phylogenetic backgrounds and ecological sources, thereby maximizing diversity within a tractable number of hosts. This experimental system, while limited in scope, provides a strategic balance between complexity and control, enabling detailed insight into the repeatability and constraints of host range evolution. At the end of the experiment, we tested for Reductions in Bacterial Growth (RBG) after phage exposure, which we take as a measure of phage virulence (Hall et al., 2011; Poullain et al., 2008; Wendling et al., 2022). In cases where phages evolved virulence against a host they could not previously infect, we interpret this as a host shift. We also included hosts that the phage could infect but only cause small reductions in bacterial growth (semi-permissive hosts) compared to other hosts found to be highly susceptible to the same phage. By evolving phage with semi-permissive hosts, we aimed to test for (1) increases in phage virulence with hosts they could already infect and (2) whether this also resulted in altered virulence against non-permissive hosts (that is, the possibility of host shifting as a ‘side-effect’ of adaptation to other hosts).

## Materials and methods

### Organisms and culture conditions

**Figure 1:**
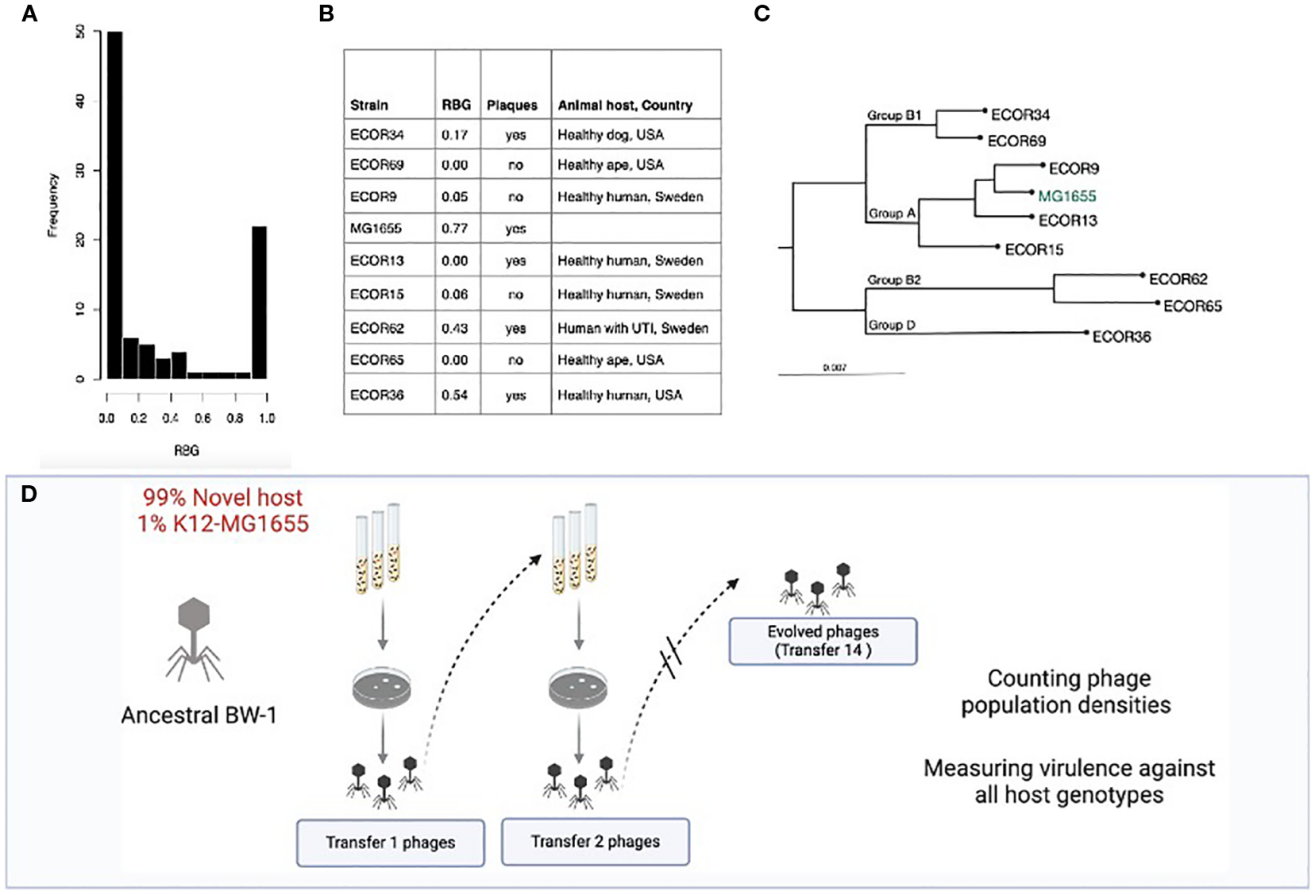
*E*. *coli* hosts used in the evolution experiment. **(A)** Histogram of Reduction in Bacterial Growth (RBG) caused by ancestral *Escherichia* phage BW-1 against 94 different natural and clinical isolates of *E*. *coli* (Allen et al., 2017). **(B)** List of bacterial hosts used in this study, with average RBG data observed in our experiments after 24h of exposure to ancestral *Escherichia* phage BW-1, plaque formation data and additional details about strain origins. **(C)** Phylogenetic tree of *E*. *coli* isolates from different sub-species groups used in this study. Details of how the tree was produced are provided as **Supplementary Information**. **(D)** Diagram representation of the evolution experiment for phage BW-1 and one bacterial host. Note that the ancestral phage was serially passaged in co-cultures containing 99% novel host (either permissive or semi-permissive) and 1% K12-MG1655. In this way, we included a selective pressure that would allow adaptation to the new host, but preventing phage extinction by including a small percentage of cells that the ancestral phage could infect.

We used different *E. coli* strains and *Escherichia* phage BW-1 (Ackermann and Krisch, 1997), a lytic T4-like phage from the *Straboviridae* family and Tevenviridae subfamily. This phage produces clear plaques (<0.1mm) and was found in a previous study to infect a relatively large number of natural and clinical *E. coli* isolates compared to other phages tested in the same study (Allen et al., 2017) (**Figure 1A**). Evidence suggests parasites with broader host ranges are more likely to infect new hosts (Cleaveland et al., 2001; Thines, 2019). Therefore, we assumed *Escherichia* phage BW-1 was a favorable candidate for host shifts. Its intermediate host range made it particularly suitable for assessing both potential for adaptation and constraints on host range expansion under selection. As bacterial hosts, we selected eight strains from the *E. coli* (ECOR) collection (Ochman and Selander, 1984). The strains varied in their level of susceptibility to ancestral *Escherichia* phage BW-1 infection, as measured by the level of Reduction in Bacterial Growth (RBG, described in more detail below) (**Figure 1B**). We included five strains where we detected little or no reduction in bacterial growth caused by ancestral *Escherichia* phage BW-1 compared to phage-free cultures (RBG equal or close to zero; **Figure 1B**). Hereafter, we refer to these strains as non-permissive hosts. For four of these strains, we also detected no evidence of plaque formation by *Escherichia* phage BW-1 when spotted on an agar lawn of the bacteria. As semi-permissive hosts, we included three strains with a significant reduction in bacterial growth caused by *Escherichia* phage BW-1. However, this reduction was at an intermediate level compared to that observed for ancestral *Escherichia* phage BW-1 across a wide range of natural and clinical isolates, as estimated in a previous study that also included the isolates used here (Allen et al., 2017). Note that the reduction in bacterial growth caused by ancestral *Escherichia* phage BW-1 on each strain in this previous study was strongly correlated with that in our experiment (*r*^2^ = 0.81, *F*_1,7_ = 29.3, *P* = 0.001). We also found that ancestral *Escherichia* phage BW-1 formed plaques on all these semi-permissive hosts. The strains were selected to maximize phylogenetic and ecological diversity within the species, while still allowing experimental tractability across replicates and time points. We chose strains from various phylogenetic subgroups of *E. coli*, as inferred previously from whole-genome sequence data (Allen et al., 2017)(**Figure 1C**). Finally, we included *E. coli* K12-MG1655 in our experiments, which is highly susceptible to *Escherichia* phage BW-1 infection (**Figure 1C**). All experiments were performed at 37 °C in lysogeny broth (LB) medium supplemented with 10mM MgSO_4_ and 10mM Tris HCL, hereafter referred to as LBMT.

### Phage evolution experiment

**Figure 2:**
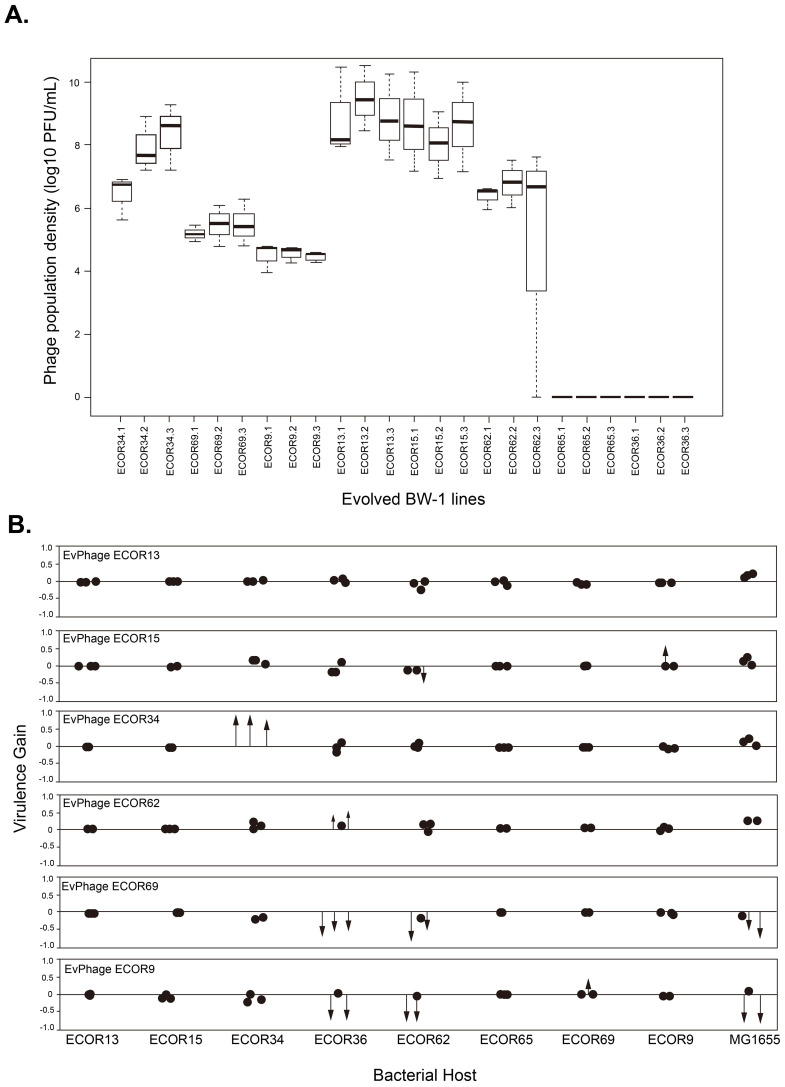
**(A)** Phage population densities of evolved *Escherichia* phage BW-1 lines at the end of the evolution experiment (Transfer 14). The name of each evolved BW-1 line (*x*-axis) indicates which bacterial host was present during the evolution experiment. Replicate selection lines are differentiated with numbers (1-3), and each box shows the results of three replicate assays. **(B)** Changes in virulence after evolving bacteriophage BW-1 on different *E. coli* hosts. Virulence gain was estimated as the difference in Reductions in Bacterial Growth (RBG) between evolved and ancestral phage for each bacterial host. Positive values indicate an increased virulence, and negative values indicate decreased virulence following evolution. Each point represents an individual evolved line. Statistically significant changes in virulence are shown as arrows, with the arrowhead marking the exact magnitude of virulence gain or loss. Non-significant changes are shown as simple dots. RGB data for this figure can be found in **Supplementary Figures S2** and **S3**.

We set up our evolution experiment to simulate a tough but realistic situation in which phages would need to evolve to infect new bacterial hosts in order to survive. To test whether host shifts (significant increase in RBG after evolution in hosts where RBG of ancestral *Escherichia* phage BW-1 equals zero) and/or high levels of virulence could be selected for, we evolved *Escherichia* phage BW-1 in non-evolving populations of each non-permissive or semi-permissive host (see diagram in **Figure 1D**). For each bacterial host (*n* = 8), we experimentally evolved three independent populations (*n* = 24 evolved phage lines). We initiated each phage line by adding 10^5^ plaque-forming units (PFU) ancestral *Escherichia* phage BW-1 particles to mixed bacterial populations (6mL cultures) consisting of 1% permissive host (*E. coli* K12-MG1655) and 99% of either non-permissive or semi-permissive host (ECOR strain) (**Figure 1D**). This initial ratio was specifically chosen to impose strong selection for host-range expansion while maintaining minimal access to a permissive host, preventing immediate phage extinction. Culturing phages in mixed bacterial populations (Benmayor et al., 2009; Borin et al., 2021) helps to maintain phage population turnover while keeping a large number of susceptible cells for any phage mutant that can infect the new host. To prevent any cumulative shift in bacterial strain frequencies, each 24-hour transfer cycle was initiated using fresh overnight cultures prepared from frozen bacterial stocks, and the 1%:99% ratio of MG1655 to test host has re-established at the start of every transfer. This ensured that any within-transfer overrepresentation of MG1655 did not carry over across passages and allowed us to preserve a consistent and reproducible selective environment for all evolving phage lines. We extracted phage after 24h (Transfer-1 phage lysates), adding 10% chloroform to each culture, vortexing for 1 min, and centrifuging at 13,000 rpm for 2 min (Buckling and Rainey, 2002). We transferred 50µl of each phage lysate to new non-evolving mixed bacterial populations (stocks from the freezer), meaning that bacterial hosts were not allowed to evolve in our experiment. We incubated and extracted phage as described above for a total of 14 transfers, which we expected to be enough time for the emergence of virulent mutants under strong selection based on previous work with other phage species (Benmayor et al., 2009; Borin et al., 2021; Hall et al., 2011). To verify phage presence in every transfer, we spotted 3.5µl of phage lysate onto a lawn of the susceptible host (*E. coli* K12-MG1655) and incubated overnight to look for clear spots or plaques. At the end of the experiment (transfer 14), we plated serial dilutions and estimated phage titers in each phage line (**Figures 1D**, **2A**). For phage lines that did not go extinct, we tested for variation in final phage population size (titer in PFU/mL) among populations evolved with different host strains. To do this, we used one-way ANOVA with titer as the response variable (mean of 3 replicate measurements for each of 3 phage lines per host strain) and host strain during the evolution experiment as a factor. We then tested for pairwise differences in phage population densities per host strain, correcting for multiple comparisons using Tukey’s Honest significant difference (**Figure 2A**).

### Measuring changes in virulence

We quantified virulence differences among the ancestral and evolved phage lines by estimating the reduction in bacterial growth (RBG) due to phage infection after 24h (Hall et al., 2011; Poullain et al., 2008; Wendling et al., 2022). We did a cross-infection experiment, testing the ancestral phage and all evolved phage lines against all hosts, including the ones they evolved with and the ones they did not. Independent bacterial populations (*n* = 6) of each host (*n* = 9) were exposed to either ancestral phage, an evolved phage line (*n* = 18) or phage-free conditions (control) in microcosms containing fresh media supplemented with phage titers adjusted to reach the same multiplicity of infection in all populations (MOI = 10). The basic setup was 10^4^ bacterial cells + 10^5^ PFU particles in volume of 100µl where at least 90% was fresh LBMT. After 24h, we measured bacterial biomass as optical density (OD24h) in each population (n=1080) using an Infinite M200 spectrophotometer (Tecan, USA). All measurements were corrected by subtracting the mean score of sterile medium (OD = 0.045). We calculated the RBG (Reduction in Bacterial Growth) values for each bacterial population in the experiment with the equation:


$$\text{ RBG}=1-\frac{OD24h\;with\;phage}{mean(OD24h\;without\;phage)}$$

For each host strain, we tested whether evolved phage populations showed altered virulence (RBG) compared to the ancestral phage using pairwise Welch’s *t*-tests, which allow for unequal variances as observed in some of our comparisons. In cases where we tested multiple evolved populations against the same control, we corrected for multiple testing by sequential Bonferroni adjustment (**Figure 2B**). Virulence gain was estimated with respect to Reductions in Bacterial Growth (RBG) of evolved phage versus ancestral phage (i.e. evolved RBG-ancestral RBG). Positive values indicate an increase in virulence after evolution, and negative values indicate reduced virulence. In **Figure 2B**, each data point represents the virulence gain for an evolved line. Statistically significant changes in virulence are shown as arrows, with the arrowhead positioned at the exact level of virulence gain or loss (**Figure 2B**; RGB data for this figure can be found in **Supplementary Figures S2** and **S3**). Non-significant changes are shown as simple dots. This visual distinction highlights where meaningful evolutionary shifts in virulence occurred.

### Estimation of genetic distance to native host range

In order to determine whether the host strains in our experiment were relatively closely or distantly related to host strains against which ancestral *Escherichia* phage BW-1 is highly virulent, we computed the average genetic distance of each non-permissive and semi-permissive host to a set of host strains found previously to be highly susceptible to *Escherichia* phage BW-1 (Allen et al., 2017). These data show that susceptibility to *Escherichia* phage BW-1 is bimodal among these isolates (**Figure 1A**). We therefore took all isolates (*n* = 94) where RBG>0.5 as being relatively highly susceptible. We then calculated the average genetic distance for each strain used in our experiment to this set of highly susceptible strains. We estimated the genetic distance between a given pair of strains as patristic difference using the *adephylo* package (Jombart et al., 2010). This is the sum of the branch lengths between the two strains, where branch length is the expected number of nucleotide changes per base pair across the 1424 loci used for phylogenetic reconstruction (**Supplementary Information S1**). For each strain used in our experiment we calculate these distances to each of the highly susceptible strains, and then take the average as a measure of genetic distance to the native host range of *Escherichia* phage BW-1. We then tested whether genetic distance to the native host range was correlated with the change in virulence over the course of selection (mean(RBGevolved)−mean(RBGancestral)) for each group of three replicates evolved with the same host). All statistical analyses were conducted in R 3.1.1 (R Core Team, 2015).

### Phage genomic extraction, sequencing, and annotation

Phage genome isolation was performed as previously described (Green and Sambrook, 2012). Briefly: 1 mL of phage stock was treated with ten µg DNaseI and 50 µg RNaseA (Roche Diagnostics; Mannheim, Germany) in the presence of MgCl2 to degrade the bacterial DNA that is still present after phage production, followed by 50 µg/mL of proteinase K (Thermo Scientific, Waltham, MA, USA), 20 mM EDTA and 0.5% SDS treatment to inactivate the DNaseI/RNaseA and to disrupt the phage capsid proteins.

Subsequently, extraction by phenol-chloroform (Carl Roth GmbH, Karlsruhe, Germany) was performed to remove debris. The nucleic acid pellet was precipitated (14,000× g, 20 min) in the presence of absolute alcohol (Merck KGaA, Darmstadt, Germany) and washed with 70% alcohol before being suspended in deionized distilled water. Nanodrop measurements (Peqlab; Erlangen, Germany) were done to determine concentration and purity (260/230 ratio). Sequencing was performed on an Illumina (San Diego, CA, USA) MiniSeq instrument. The Nextera Flex DNA library kit (Illumina) was used for library preparation. Long reads were generated using an Oxford Nanopore MinION (Oxford, UK) device with an R.9.4.1 flow cell. The latter library was prepared with Rapid barcoding. Sequence quality was examined with FastQC (https://www.bioinformatics.babraham.ac.uk/projects/fastqc/). Low-quality reads and adapter removal from the paired ends for all sequences were filtered by Trim Galore v. 0.6.6 (https://www.bioinformatics.babraham.ac.uk/projects/trim_galore/) using a Phred quality cutoff of 30. Then, human reads were discarded by mapping them against the Genome Consortium Human Build 38(GRCh38; Schneider et al., 2017) using Bowtie2 v. 2.4.2 (Langmead and Salzberg, 2012) with default parameters. For the *de novo* assembly, metaSPAdes v. 3.15.0 (Bankevich et al., 2012; Nurk et al., 2017) with default parameters was executed as recommended in previous publications (Sutton et al., 2019). The resulting contigs were annotated using VIGA 0.11.1 (Gonzalez-Tortuero et al., 2022; González-Tortuero et al., 2018) using the RefSeq Viral Database (Brister et al., 2015), the Viral Orthologs Groups(VOGs; Marz et al., 2014), the Viral DataBase (RVDB v. 21.0; Bigot et al., 2020) and the Prokaryotic virus Remote Homologous Groups (PHROGS v. 4; Terzian et al., 2021) for the functional prediction. Variant calling between the evolved Escherichia phages and the ancestral one was performed using Snippy v. 4.6 (https://github.com/tseemann/snippy).

### Comparative genomic analysis and visualization

To investigate the potential regulatory or functional impact of SNP 164264, a 1,000 bp region centered on the SNP was extracted from the assembled BW1 genome and aligned to the homologous region in the Escherichia phage T4 (reference genome NC_000866.4). Protein-coding open reading frames (ORFs) in the BW1 region were predicted using VIGA v.0.12.0 (González-Tortuero et al., 2018), by scanning all putative reading frames using the bacterial genetic code (11) and a minimum threshold of 60bp for ORF length. Gene annotations for T4 were retrieved from GenBank and the noncoding RNAs rnaC and rnaD (also known as species 1 and 2 RNAs) were mapped based on the coordinates described by Miller et al. (2003). A comparative figure was generated to visualize the gene architecture, the SNP position, and the local sequence context. Features displayed include directional gene arrows, codon context (highlighting the T-C transition at the SNP), putative BW-1 specific ORFs, and the location of predicted regulatory elements. All visualizations were constructed using SnapGene v. 8.1.1 and in Python v. 3.12.1 using the matplotlib 3.8.2 library.

### Virulence assay against ECOR collection strains

To test whether gains in virulence after evolution affected virulence patterns towards other *E. coli* strains, we performed a fully factorial cross infection experiment, using the evolved phages where we observed a consistent increase in virulence across the 3 replicate lines against all members of the ECOR collection. We tested virulence differences of the evolved phages against all 72 strains in ECOR collection as compared to the virulence observed in the ancestral phage. The overnight cultures (corresponding to approx. 10^8^ CFU/mL) were then mixed with either ancestral phage, an evolved phage line (*n* = 3) or phage-free conditions (control) at MOI = 10 (10^5^ PFU/mL) after diluted to 10^4^ CFU/mL and incubated at 37°C. After 24h, the bacterial biomass was measured as optical density (OD_600_24h) in each population (3 replicates per experimental condition for an overall total of n=1080) using an Infinite M200 spectrophotometer (Tecan, USA). All measurements were corrected by subtracting the mean score of sterile media (OD = 0.0495). The reduction in bacterial growth (RBG values) were calculated for each population, as mentioned before. For each host strain, we tested whether evolved phage populations showed altered virulence (RBG) compared to the ancestral phage using pairwise Welch’s *t*-tests, which allow for unequal variances as observed in some of our comparisons. In cases where we tested multiple evolved populations against the same control, we corrected for multiple testing by sequential Bonferroni adjustment.

## Results

### Variation in phage population densities at the end of the evolution experiment

Evolving with new hosts had a significant effect on phage population densities by the end of the experiment. Despite continued mixed-host culturing until the final transfer in all phage lines (*n* = 24), we observed phage extinction in lines that evolved with one non-permissive host (ECOR65) and one semi-permissive host (ECOR36) (**Figure 2A**). Extinctions happened early in the evolution experiment. We had no phage detection after transfer 2 in all phage lines that evolved with ECOR36 and two phage lines that evolved with ECOR65 (the third phage line went extinct at transfer 3). As for the phage lines where phage did not go extinct, we found significant differences in average phage population densities depending on the host they evolved with (*F*_5,12_ = 24.42, *P*<0.0001 by one-way ANOVA). Phage lines that evolved with ECOR9, ECOR69, and ECOR62 had significantly lower average phage population densities than phage lines that evolved with ECOR15 and ECOR13 (Tukey HSD for all pairwise comparisons *P*<0.05) (**Figure 2A**).

### Virulence increases in evolved phages are rare and confined to semi-permissive hosts, with host shifts remaining unconfirmed

The majority of evolved phage lines did not show significant changes in virulence compared to the ancestral phage. Specifically, in 12 out of the 18 cases tested, we observed no significant differences in relative bacterial growth (RBG) (P > 0.05 in all cases by Welch’s t-test corrected with sequential Bonferroni; see black dots in **Figure 2B**; RBG values in **Supplementary Figures S2**, **S3**). However, we identified some cases where virulence increased, and these gains were primarily observed in phages that evolved with semi-permissive hosts. Notably, phage lines evolved with the semi-permissive host ECOR34 showed substantial increases in virulence, with an average RBG value of 0.98 ± 0.02 compared to 0.17 for the ancestral phage (P < 0.05; see red arrows in **Figure 2B**; **Supplementary Figure S3**). By contrast, none of the phages evolved with non-permissive hosts exhibited any significant gains in virulence (**Figure 2B**; **Supplementary Figure S2**).

Additionally, we found one intriguing data point suggesting a possible host shift. Specifically, phage lines evolved with ECOR69 appeared to increase infectivity based on RBG data, but when we spot-plated these phage lines on ECOR69 agar plates, they failed to form plaques, providing no evidence of successful infection on this host. Conversely, phage lines evolved with ECOR15 were able to form plaques on ECOR15 agar plates despite showing no significant increase in virulence in liquid culture (P > 0.05 in all cases). These results highlight that while virulence increases were rare and primarily restricted to semi-permissive hosts, liquid culture infectivity does not necessarily correlate with plaque formation on agar surfaces.

First, we asked whether evolved phage lines were more virulent towards their corresponding bacterial host (the host strain they were exposed to during experimental evolution, shown in **Figure 2B**). We found significant gains in virulence (*P*<0.05 in pairwise comparisons against the ancestral phage using Welch’s *t*-test corrected with sequential Bonferroni) in six phage lines corresponding to three hosts (see red arrows in **Figure 2B**). None of the phage lines that evolved with non-permissive host had significant gains in virulence. However, in one specific case (evolution with the semi-permissive host ECOR34) phage lines showed a considerable gain in virulence (average RBG for evolved phage lines=0.98 ± 0.02; RBG for the ancestral phage=0.17) (**Supplementary Figure S3**), highlighting that changes can occur under permissive conditions. For the remaining phage lines (*n* = 12) we observed no significant virulence differences from the ancestor (*P*>0.05 in all cases by Welch’s *t*-test corrected with sequential Bonferroni) (see black dots in **Figure 2B** and RGB values in S2&S3). In short, our RBG data shows that while phage BW-1 can increase virulence under certain permissive conditions (as seen with host ECOR34), it did not evolve the ability to infect any of the non-permissive hosts tested, suggesting that full host shifts remain unlikely in this system.

### Adaptation to one host can be costly in terms of virulence profiles towards other hosts

We further asked whether evolution influenced phage virulence towards hosts other than the ones encountered during the evolution experiment. For two semi-permissive hosts (ECOR36 & ECOR62) (**Figure 2B**; **S3**), multiple evolved phage lines that had been evolved with other hosts had altered virulence relative to the ancestor, and for two non-permissive hosts (ECOR9 & ECOR69) (**Figure 2B**; **S2**), we observed small increases in virulence in a single evolved phage line. Specifically, we observed that replicate phage lines that evolved with hosts ECOR9 and ECOR69 (at least two phage lines in each case) lost virulence against both hosts ECOR36 (mean RBG for all phage lines different from an ancestor that evolved with host ECOR9 = 0.211 ± 0.333, with host ECOR69 = 0.008 ± 0.003 *vs* mean RBG ancestral phage=0.544) and ECOR62 (mean RBG for all phage lines different from ancestor that evolved with host ECOR9 = 0.137 ± 0.238, with host ECOR69 = 0.101 ± 0.090 *vs* mean RBG ancestral phage=0.434). In comparison, phage lines that evolved with ECOR62 (2 out of 3 phage lines) had significant gains in virulence against ECOR36 (mean RBG for all phage lines different from an ancestor that evolved with host ECOR62 = 0.867 ± 0.121, *vs* RBG ancestral phage=0.544) (**Supplementary Figure S3**).

We also tested for changes in virulence against the permissive host K12-MG1655 (**Supplementary Figure S4**), finding that phage lines that evolved with ECOR9 and ECOR69 (2 out of 3 phage lines in both cases) had lost virulence (RBG) as compared to the ancestral phage (mean RBG for all phage lines different from an ancestor that evolved with host ECOR9 = 0.303 ± 0.465, with host ECOR69 = 0.379 ± 0.327 *vs* mean RBG ancestral phage= 0.767). In summary, phage lines that evolved with the non-permissive host ECOR69 did not gain the ability to infect the new host and still, paid the expense of losing virulence towards multiple hosts. By contrast, phages that had adapted to increase their virulence on the semi-permissive host ECOR34 did not incur a reduction in virulence on other hosts. (**Supplementary Figures S2**, **S3**).

### No evidence that adaptation is more likely when hosts are closely related to the native host range

**Figure 3:**
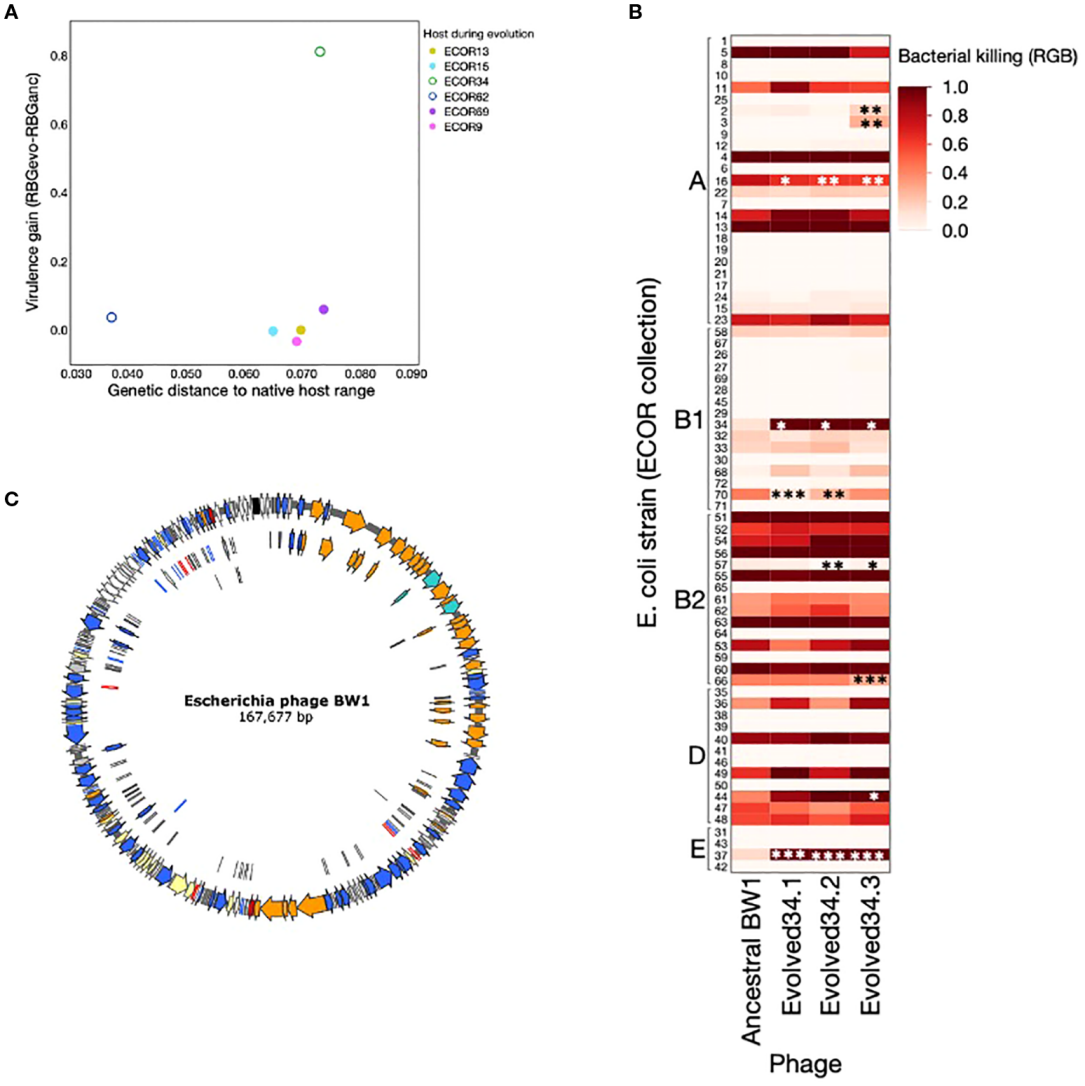
**(A)** Virulence gain (difference between average RBG for evolved phage lines and RBG for ancestral *Escherichia* phage BW-1) as a function of the genetic distance to native host range (calculated as described in methods). Each dot represents the average virulence gain for the three independent lines evolved with each host. Phage lines evolved with non-permissive hosts are shown as full circles, and phage lines evolved with semi-permissive hosts are shown as empty circles. **(B)** Phages evolved with ECOR34 tested against a set of 72 *E*. *coli* strains from the ECOR collection. Asterisks indicate cases where the evolved phage shows a statistically significant difference in virulence compared to the ancestral BW1 phage. This figure shows that increased virulence toward ECOR34 does not lead to widespread changes in virulence across other hosts—most strains show no significant change, and where changes do occur, they are primarily losses of virulence (except ECOR37). **(C)** Genome annotation of Escherichia phage BW-1. Colors indicate gene function based on viral gene classification proposed by Moura de Souza et al. (2021). Orange: structural, turquis: packaging, blue: DNA metabolism, red: lysis, yellow: regulation, white: hypothetical, black: tRNAs, grey: miscellaneous. See annotation details in **Supplementary Table S1**).

The only case where we found evidence of adaptation resulting in increased virulence (ECOR34) is the strain with the highest genetic distance to the set of highly susceptible host isolates identified in a previous study (Allen et al., 2017; **Supplementary Figure S1**) (**Figure 3A**). This was also true when we used alternative cut-off values for classifying host isolates as highly susceptible (tested for RBG = 0.3, 0.7 & 0.9). This provides no support for the idea that adaptation resulting in increased virulence is more likely in host strains that are closely related to the current host range of the pathogen.

### Convergent evolution in phage adaptation: a regulatory SNP drives adaptation to semi-permissive *E. coli* host

**Figure 4:**
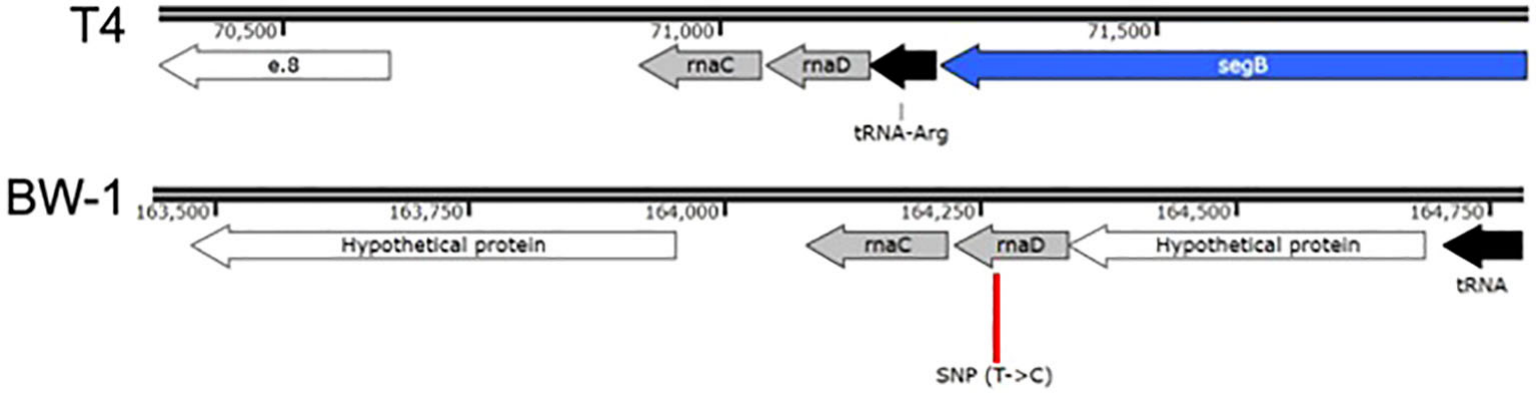
Genomic context of the convergent SNP (position 164264) found in all phage lines evolved with ECOR34. This SNP lies in a noncoding intergenic region on the reverse strand of the BW1 genome, between two hypothetical protein-coding genes. Comparative analysis reveals homology with a well-described region of phage T4 that contains two small noncoding RNAs: rnaC and rnaD (Miller et al., 2003). In BW1, rnaC (~138 bp) is more conserved, while rnaD (~118 bp) is more degenerated relative to T4. The SNP occurs within the 3′ region of rnaD, at approximately position 41 of the predicted transcript.

To gain deeper insight into the genetic changes underlying increased phage virulence, we performed whole-genome sequencing on phages evolved with the semi-permissive host ECOR34. Based on prior studies of T4-like phages such as BW-1, we anticipated identifying multiple polymorphisms, particularly in genes involved in host specificity, such as those encoding the distal regions of the long tail fibers (Miller et al., 2003; Taslem Mourosi et al., 2022). The C-terminal region of gp37, known to be hypervariable (Hashemolhosseini et al., 1994; Montag et al., 1990; Taslem Mourosi et al., 2022)and implicated in host range expansion, was of particular interest (Chen et al., 2017). However, sequencing revealed surprisingly limited genetic changes across three independent evolution experiments. A single, shared single-nucleotide polymorphism (SNP) was consistently identified in all evolved populations, located within a non-coding regulatory region between two hypothetical genes (**Figure 4**).

Further inspection revealed that this region corresponds to the reverse strand and aligns with two adjacent noncoding RNAs described in phage T4: rnaC and rnaD (Miller et al., 2003). These small RNAs, though of unknown function, are transcribed sequentially and occupy the genomic coordinates 164080–164336 in our phage BW1, with *rnaC* (~138 bp) located upstream of *rnaD* (~118 bp). The SNP lies within the 3′ end of *rnaD*, approximately at position 41 of the predicted transcript. While *rnaC* appears to be well conserved (90% conservation), *rnaD* is more degenerated in BW1 compared to its T4 counterpart. RNAfold predictions suggested that the BW1 rnaD region may adopt a somewhat more stable secondary structure than the corresponding region in T4. The presence of the SNP did not appear to drastically alter the overall fold in these preliminary predictions, but its potential impact on local stability remains to be fully tested. No additional consistent mutations were detected across the three populations, suggesting that adaptive changes during co-evolution with ECOR34 were constrained and focused on this regulatory locus (See **Supplementary Tables S2**-**S4**). While the functional implications of this mutation remain to be fully elucidated, its convergence across all replicates strongly suggests a potential role in the phage’s adaptation to the semi-permissive host ECOR34. This finding highlights the specificity of genomic changes associated with host shifts under controlled laboratory conditions.

## Discussion

We used a controlled experimental framework to investigate the evolution of bacteriophage host shifts and changes in virulence. Our study focused on a single phage BW1 and a carefully selected panel of eight *E. coli* strains, allowing us to probe the dynamics of host shifts with high experimental resolution. While this narrow scope limits broad generalizations, it enabled us to test evolutionary outcomes under tightly controlled and reproducible conditions.

Our finding that evolved phages had increased virulence in just a limited number of cases is consistent with the overall expectation that although host shifts can happen, they are rare and limited events (Benmayor et al., 2009; De Sordi et al., 2017; Hall et al., 2011; Marchi et al., 2023; Scanlan et al., 2013). Of the hosts where we observed adaptation, changes were only found in the semi-permissive hosts, demonstrating that phages primarily adapt to hosts they can already infect to some degree. Whole-genome sequencing of the evolved phage lines with ECOR34 revealed a single shared SNP located in a non-coding intergenic region of the reverse strand. Further analysis revealed that this region aligns with the rnaC–rnaD noncoding RNA cluster described in phage T4 (Miller et al., 2003). These two adjacent ncRNAs have unknown functions but are thought to play regulatory roles. In BW1, *rnaC* appears to be relatively conserved, whereas *rnaD* is more degenerated. The SNP in question lies within the 3′ end of *rnaD*, around position 41 of the predicted transcript. Using RNAfold, we found that the rnaD region in BW1 (with and without the SNP) appears to adopt more stable secondary structures than the corresponding region in phage T4, which is more structurally flexible. This increased structural stability in BW1 may reflect reduced regulatory flexibility, potentially affecting the timing or expression of downstream genes. We emphasize that these predictions are exploratory, and the precise structural and functional consequences of this SNP remain to be fully determined.

The lack of host shifts might be explained by their cost. Phages that evolved with ECOR69, when compared to the ancestral phage, displayed loss of virulence towards other hosts. These results suggest that a host shift might be more costly than beneficial in complex bacterial communities. Such hypothesis might explain the results of our experiment: acquiring a mutation that allows infection of other hosts may be so costly in terms of virulence that such mutants are not able to replicate fast enough in our experiments and therefore we were unable to detect them. Cost expressed as reduced virulence have been observed previously in bacteriophages infecting *Pseudomonas fluorescens* (Poullain et al., 2008; Wang et al., 2024). The genomic constraint observed in our WGS data further supports the idea that evolutionary paths toward broader host range may be both rare and deeply bounded by trade-offs or lack of mutational accessibility.

Our data also suggests that host shifts are not simply determined by genetic similarity of the new host to the pathogen’s native host. Although our data do not have sufficient statistical power (due to a limited number of hosts) to test this definitively, it was not the case that hosts closely related to the native host range were easier to adapt to. In fact, the host where we observed adaptation (ECOR34) is the most distant to the native host range. An alternative hypothesis is that adaptation to new hosts is driven by the presence of specific components, like phage receptors (Bertozzi Silva et al., 2016; Burmeister et al., 2021) or plasmids (Jalasvuori et al., 2011; Shan et al., 2023), which can be transmitted horizontally (Tzipilevich et al., 2017) and independently of phylogeny. The observed SNP in a regulatory region across all ECOR34-adapted phages may reflect subtle tuning of gene expression in response to these specific host factors, rather than structural changes in host-recognition proteins.

Our results have several implications for phage therapy. Our data support the prevailing view that host shifts are rare events due to high specificity of phages, and suggest that the risk of unintended phage activity on commensal bacteria is low, even under experimental conditions designed to promote such shifts. Nevertheless, host shifts are still possible events, and our data show that adaptation to one host can result in either loss or gain of virulence towards different hosts. A recent study with *in vitro* and *in vivo* experiments using mice (De Sordi et al., 2017) found that intermediate hosts from gut microbiota are important for phage host shifts, but even in these cases, host shifts were observed only in 20% of the cases. These complementary findings imply that future studies looking at the safety of phages need to incorporate the complexity of the gut microbiota. A future study could perform *in vitro* evolution experiments like ours but using greater numbers of intermediate hosts (mixed bacterial communities) in the experimental regime.

It is important to note, however, that the virulence assays used in our study rely on RGB signal reduction in liquid cultures, which may reflect not only bacterial lysis but also general growth inhibition or metabolic suppression. This contrasts with traditional agar plate assays, which provide a more direct measure of lytic activity. While our approach offers a scalable and high-throughput alternative, it is limited in its ability to distinguish between these different modes of bacterial suppression. Previous authors have noted that observations of phage-bacteria interactions in liquid and on agar surfaces sometimes differ (Hyman and Abedon, 2010; Koskella and Meaden, 2013). Future studies could address this limitation by conducting comparative virulence analyses using both agar plate and liquid culture methods. This would help clarify whether observed reductions in bacterial growth are due to true lysis or other inhibitory effects. This is an important distinction, especially considering that a phage might still reduce bacterial growth despite being unable to lyse the host effectively through its ancestral mechanisms. Additionally, characterizing the physiological and genetic mechanisms that enable or constraint host shifts, including regulatory regions like the one identified in our study, could help map the distribution of potential host-switching pathways across the microbiota. Identifying key elements such as phage receptors or mobile plasmids, and assessing their prevalence and transferability, would provide valuable insights into the dynamics and risks of host range evolution in therapeutic contexts.

## Data Availability

The datasets generated and analyzed in this study are available in the Zenodo repository at https://zenodo.org/records/17353398.

## References

[B1] Ackermann H.-W. Krisch H. M. (1997). A catalogue of T4-type bacteriophages. Arch. Virol. 142, 2329–2345. doi: 10.1007/s007050050246, PMID: 9672598

[B2] Allen R. C. Pfrunder-Cardozo K. R. Meinel D. Egli A. Hall A. R. (2017). Associations among antibiotic and phage resistance phenotypes in natural and clinical Escherichia coli isolates. MBio 8, e01341–17. doi: 10.1128/mBio.01341-17, PMID: 29089428 PMC5666156

[B3] Angermeyer A. Hays S. G. Nguyen M. H. T. Johura F. T. Sultana M. Alam M. . (2021). Evolutionary sweeps of subviral parasites and their phage host bring unique parasite variants and disappearance of a phage CRISPR-cas system. MBio 13, e0308821. doi: 10.1128/mbio.03088-21, PMID: 35164562 PMC8844924

[B4] Bankevich A. Nurk S. Antipov D. Gurevich A. A. Dvorkin M. Kulikov A. S. . (2012). SPAdes: A new genome assembly algorithm and its applications to single-cell sequencing. J. Comput. Biol. 19, 455–477. doi: 10.1089/cmb.2012.0021, PMID: 22506599 PMC3342519

[B5] Benmayor R. Hodgson D. J. Perron G. G. Buckling A. (2009). Host mixing and disease emergence. Curr. Biol. 19, 764–767. doi: 10.1016/j.cub.2009.03.023, PMID: 19375316 PMC7126095

[B6] Bertozzi Silva J. Storms Z. Sauvageau D. (2016). Host receptors for bacteriophage adsorption Downloaded from. FEMS Microbiol. Lett. 363, fnw002. Available online at: http://femsle.oxfordjournals.org/ (Accessed September 02, 2018). 26755501 10.1093/femsle/fnw002

[B7] Bigot T. Temmam S. Pérot P. Eloit M. (2020). RVDB-prot, a reference viral protein database and its HMM profiles. F1000Research 8, 1–12. doi: 10.12688/f1000research.18776.2, PMID: 32983411 PMC7492780

[B8] Bohannan B. J. M. Lenski R. E. (2000). Linking genetic change to community evolution: insights from studies of bacteria and bacteriophage. Ecol. Lett. 3, 362–377. doi: 10.1046/j.1461-0248.2000.00161.x

[B9] Borin J. M. Avrani S. Barrick J. E. Petrie K. L. Meyer J. R. (2021). Coevolutionary phage training leads to greater bacterial suppression and delays the evolution of phage resistance. Proc. Natl. Acad. Sci. United States America 118, e2104592118. doi: 10.1073/pnas.2104592118, PMID: 34083444 PMC8201913

[B10] Brister J. R. Ako-Adjei D. Bao Y. Blinkova O. (2015). NCBI viral Genomes resource. Nucleic Acids Res. 43, D571–D577. doi: 10.1093/nar/gku1207, PMID: 25428358 PMC4383986

[B11] Buckling A. Rainey P. B. (2002). 2002 The role of parasites in sympatric and allopatric host diversification.pdf. Nature 420, 496–499. doi: 10.1038/nature01164, PMID: 12466840

[B12] Burmeister A. R. Sullivan R. M. Gallie J. Lenski R. E. (2021). Sustained coevolution of phage lambda and escherichia coli involves inner- as well as outer-membrane-Defences and counter-Defences. Microbiol. (United Kingdom) 167, 001063. doi: 10.1099/MIC.0.001063, PMID: 34032565 PMC8290101

[B13] Chen M. Zhang L. Abdelgader S. A. Yu L. Xu J. Yao H. . (2017). Alterations in gp37 expand the host range of a T4-like phage. Appl. Environ. Microbiol. 83, e01576–17. doi: 10.1128/AEM.01576-17, PMID: 28939606 PMC5691408

[B14] Cleaveland S. Laurenson M. K. Taylor L. H. (2001). Diseases of humans and their domestic mammals: Pathogen characteristics, host range and the risk of emergence. Philos. Trans. R. Soc. B 356, 991–999. doi: 10.1098/rstb.2001.0889, PMID: 11516377 PMC1088494

[B15] De Sordi L. Khanna V. Debarbieux L. (2017). The gut microbiota facilitates drifts in the genetic diversity and infectivity of bacterial viruses. Cell Host Microbe 22, 801–808.e3. doi: 10.1016/j.chom.2017.10.010, PMID: 29174401

[B16] Gonzalez-Tortuero E. Krishnamurthi R. Goodhead I. Allison H. James C. (2022). Improving phage genome annotation to understand phage biology: the case of Pseudomonas aeruginosa LES prophages. Access Microbiol. 4, 277509. doi: 10.1099/acmi.ac2021.po0318

[B17] González-Tortuero E. Sean Sutton T. D. Velayudhan V. Shkoporov A. N. Draper L. A. Stockdale S. R. . (2018). VIGA: A sensitive, precise and automatic *de novo* VIral Genome Annotator. BioRxiv. doi: 10.1101/277509

[B18] Green M. R. Sambrook J. (2012). “ Molecular cloning: A laboratory manual,” in Dong wu xue yan jiu = Zoological research/”Dong wu xue yan jiu” bian ji wei yuan hui bian ji, vol. 1. (Yunnan, China: Cold Spring Harbor Laboratory Press). doi: 10.3724/sp.j.1141.2012.01075, PMID: 22345012

[B19] Hall A. R. Scanlan P. D. Buckling A. (2011). Bacteria-phage coevolution and the emergence of generalist pathogens. Am. Nat. 177, 44–53. doi: 10.1086/657441, PMID: 21117957

[B20] Hashemolhosseini S. Montag D. Kramer L. Henning U. (1994). Determinants of receptor specificity of coliphages of the T4 family. A chaperone alters the host range. J. Mol. Biol. 241, 524–533. doi: 10.1006/jmbi.1994.1529, PMID: 8057378

[B21] Hyman P. Abedon S. T. (2010). “ Bacteriophage host range and bacterial resistance,” in Advances in Applied Microbiology, 1st ed, vol. 70. (Amsterdam, The Netherlands: Elsevier Inc). doi: 10.1016/S0065-2164(10)70007-1, PMID: 20359459

[B22] Jalasvuori M. Friman V. P. Nieminen A. Bamford J. K. H. Buckling A. (2011). Bacteriophage selection against a plasmid-encoded sex apparatus leads to the loss of antibioticresistance plasmids. Biol. Lett. 7, 902–905. doi: 10.1098/rsbl.2011.0384, PMID: 21632619 PMC3210665

[B23] Jombart T. Balloux F. Dray S. (2010). adephylo: New tools for investigating the phylogenetic signal in biological traits. Bioinformatics 26, 1907–1909. doi: 10.1093/bioinformatics/btq292, PMID: 20525823

[B24] Kaper J. B. Nataro J. P. Mobley H. L. T. (2004). Pathogenic escherichia coli. Nat. Rev. Microbiol. 2, 123–140. doi: 10.1038/nrmicro818, PMID: 15040260

[B25] Kim M. J. Bae H. E. Kwon S. Park M. K. Yong D. Kang M. J. . (2023). Phage-targeting bimetallic nanoplasmonic biochip functionalized with bacterial outer membranes as a biorecognition element. Biosensors Bioelectronics 238, 115598. doi: 10.1016/j.bios.2023.115598, PMID: 37597282

[B26] Koskella B. Hernandez C. A. Wheatley R. M. (2022). Understanding the impacts of bacteriophage viruses: from laboratory evolution to natural ecosystems. Annu. Rev. Virol. 9, 57–78. doi: 10.1146/annurev-virology-091919-075914, PMID: 35584889

[B27] Koskella B. Meaden S. (2013). Understanding bacteriophage specificity in natural microbial communities. Viruses 5, 806–823. doi: 10.3390/v5030806, PMID: 23478639 PMC3705297

[B28] Langmead B. Salzberg S. L. (2012). Fast gapped-read alignment with Bowtie 2. Nat. Methods 9, 357–359. doi: 10.1038/nmeth.1923, PMID: 22388286 PMC3322381

[B29] Lee C.-Y. (2024). Exploring potential intermediates in the cross-species transmission of influenza A virus to humans. Viruses 16, 1129. doi: 10.3390/v16071129, PMID: 39066291 PMC11281536

[B30] Li L. Wu Y. Ma D. Zhou Y. Wang L. Han K. . (2022). Isolation and characterization of a novel Escherichia coli phage Kayfunavirus ZH4. Virus Genes 58, 448–457. doi: 10.1007/s11262-022-01916-6, PMID: 35716226

[B31] Liu W. Li Y. Learn G. H. Rudicell R. S. Robertson J. D. Keele B. F. . (2010). Origin of the human malaria parasite Plasmodium falciparum in gorillas. Nature 467, 420–425. doi: 10.1038/nature09442, PMID: 20864995 PMC2997044

[B32] Longdon B. Brockhurst M. A. Russell C. A. Welch J. J. Jiggins F. M. (2014). The evolution and genetics of virus host shifts. PLoS Pathog. 10, e1004395–8. doi: 10.1371/journal.ppat.1004395, PMID: 25375777 PMC4223060

[B33] Longdon B. Hadfield J. D. Webster C. L. Obbard D. J. Jiggins F. M. (2011). Host phylogeny determines viral persistence and replication in novel hosts. PLoS Pathog. 7, e1002260. doi: 10.1371/journal.ppat.1002260, PMID: 21966271 PMC3178573

[B34] Marchi J. Zborowsky S. Debarbieux L. Weitz J. S. (2023). The dynamic interplay of bacteriophage, bacteria and the mammalian host during phage therapy. IScience 26, 106004. doi: 10.1016/j.isci.2023.106004, PMID: 36818291 PMC9932479

[B35] Marz M. Beerenwinkel N. Drosten C. Fricke M. Frishman D. Hofacker I. L. . (2014). bioinformatics%2Fbtu105.pdf. (Oxford, England), 1–7. 10.1093/bioinformatics/btu105 PMC7110044 24590443

[B36] Michel A. Clermont O. Denamur E. Tenaillon O. (2010). Bacteriophage PhiX174’s ecological niche and the flexibility of its escherichia coli lipopolysaccharide receptor. Appl. Environ. Microbiol. 76, 7310–7313. doi: 10.1128/AEM.02721-09, PMID: 20833781 PMC2976268

[B37] Miller E. S. Kutter E. Mosig G. Arisaka F. Kunisawa T. Rüger W. (2003). Bacteriophage T4 genome. Microbiol. Mol. Biol. Rev. 67, 86–156. doi: 10.1128/mmbr.67.1.86-156.2003, PMID: 12626685 PMC150520

[B38] Mirzaei M. K. Maurice C. F. (2017). Ménage à trois in the human gut: Interactions between host, bacteria and phages. Nat. Rev. Microbiol. 15, 397–408. doi: 10.1038/nrmicro.2017.30, PMID: 28461690

[B39] Mollentze N. Streicker D. G. (2020). Viral zoonotic risk is homogenous among taxonomic orders of mammalian and avian reservoir hosts. Proc. Natl. Acad. Sci. United States America 117, 9423–9430. doi: 10.1073/pnas.1919176117, PMID: 32284401 PMC7196766

[B40] Montag D. Hashemolhosseini S. Henning U. (1990). Receptor-recognizing proteins of T-even type bacteriophages. The receptor-recognizing area of proteins 37 of phages T4 TuIa and TuIb. J. Mol. Biol. 216, 327–334. doi: 10.1016/S0022-2836(05)80324-9, PMID: 2147721

[B41] Moura de Souza J. A. Pfeifer E. Touchon M. Rocha E. P. C. (2021). Causes and consequences of bacteriophage diversification via genetic exchanges across lifestyles and bacterial taxa. Mol. Biol. Evol. 38, 2497–2512. 10.1093/molbev/msab044, PMID: 33570565 PMC8136500

[B42] Nurk S. Meleshko D. Korobeynikov A. Pevzner P. A. (2017). MetaSPAdes: A new versatile metagenomic assembler. Genome Res. 27, 824–834. doi: 10.1101/gr.213959.116, PMID: 28298430 PMC5411777

[B43] Ochman H. Selander R. K. (1984). Standard reference strains of Escherichia coli from natural populations. J. Bacteriology 157, 690–693. doi: 10.1128/jb.157.2.690-693.1984, PMID: 6363394 PMC215307

[B44] Poullain V. Gandon S. Brockhurst M. A. Buckling A. Hochberg M. E. (2008). The evolution of specificity in evolving and coevolving antagonistic interactions between a bacteria and its phage. Evolution 62, 1–11. doi: 10.1111/j.1558-5646.2007.00260.x, PMID: 18005153

[B45] R Core Team (2015). R: A Language and Environment for Statistical Computing (Vienna, Austria: R Foundation for Statistical Computing). 2014. R Foundation for Statistical Computing. *R Foundation for Statistical Computing, Vienna, Austria.*, 2, 2019.

[B46] Scanlan P. D. Hall A. R. Burlinson P. Preston G. Buckling A. (2013). No effect of host-parasite co-evolution on host range expansion. J. Evolutionary Biol. 26, 205–209. doi: 10.1111/jeb.12021, PMID: 23167752

[B47] Schneider V. A. Graves-Lindsay T. Howe K. Bouk N. Chen H. C. Kitts P. A. . (2017). Evaluation of GRCh38 and *de novo* haploid genome assemblies demonstrates the enduring quality of the reference assembly. Genome Res. 27, 849–864. doi: 10.1101/gr.213611.116, PMID: 28396521 PMC5411779

[B48] Seal S. Dharmarajan G. Khan I. (2021). Evolution of pathogen tolerance and emerging infections: A missing experimental paradigm. ELife 10, 68874. doi: 10.7554/eLife.68874, PMID: 34544548 PMC8455132

[B49] Shan X. Szabo R. E. Cordero O. X. (2023). doi: 10.1038/s41467-023-37512-x, PMID: 37041135 PMC10090143

[B50] Sharp P. M. Hahn B. H. (2010). The evolution of HIV-1 and the origin of AIDS. Philos. Trans. R. Soc. B 365, 2487–2494. doi: 10.1098/rstb.2010.0031, PMID: 20643738 PMC2935100

[B51] Shkoporov A. N. Hill C. (2019). Bacteriophages of the human gut: the “Known unknown” of the microbiome. Cell Host Microbe 25, 195–209. doi: 10.1016/j.chom.2019.01.017, PMID: 30763534

[B52] Siniagina M. N. Markelova M. I. Boulygina E. A. Laikov A. V. Khusnutdinova D. R. Abdulkhakov S. R. . (2021). Diversity and adaptations of escherichia coli strains: Exploring the intestinal community in crohn’s disease patients and healthy individuals. Microorganisms 9, 1299. doi: 10.3390/microorganisms9061299, PMID: 34203637 PMC8232093

[B53] Sorek R. Kunin V. Hugenholtz P. (2008). CRISPR - A widespread system that provides acquired resistance against phages in bacteria and archaea. Nat. Rev. Microbiol. 6, 181–186. doi: 10.1038/nrmicro1793, PMID: 18157154

[B54] Sutton T. D. S. Clooney A. G. Ryan F. J. Ross R. P. Hill C. (2019). Choice of assembly software has a critical impact on virome characterisation. Microbiome 7, 1–15. doi: 10.1186/s40168-019-0626-5, PMID: 30691529 PMC6350398

[B55] Taslem Mourosi J. Awe A. Guo W. Batra H. Ganesh H. Wu X. . (2022). Understanding bacteriophage tail fiber interaction with host surface receptor: the key “Blueprint” for reprogramming phage host range. Int. J. Mol. Sci. 23, 12146. doi: 10.3390/ijms232012146, PMID: 36292999 PMC9603124

[B56] Tenaillon O. Skurnik D. Picard B. Denamur E. (2010). The population genetics of commensal Escherichia coli. Nat. Rev. Microbiol. 8, 207–217. doi: 10.1038/nrmicro2298, PMID: 20157339

[B57] Terzian P. Olo Ndela E. Galiez C. Lossouarn J. Pérez Bucio R. E. Mom R. . (2021). PHROG: Families of prokaryotic virus proteins clustered using remote homology. NAR Genomics Bioinf. 3, 1–12. doi: 10.1093/nargab/lqab067, PMID: 34377978 PMC8341000

[B58] Thines M. (2019). An evolutionary framework for host shifts – jumping ships for survival. New Phytol. 224, 605–617. doi: 10.1111/nph.16092, PMID: 31381166

[B59] Tzipilevich E. Habusha M. Ben-Yehuda S. (2017). Acquisition of phage sensitivity by bacteria through exchange of phage receptors. Cell 168, 186–199.e12. doi: 10.1016/j.cell.2016.12.003, PMID: 28041851

[B60] Walsh S. K. Imrie R. M. Matuszewska M. Paterson G. K. Weinert L. A. Hadfield J. D. . (2023). The host phylogeny determines viral infectivity and replication across Staphylococcus host species. PLoS Pathog. 19, e1011433. doi: 10.1371/journal.ppat.1011433, PMID: 37289828 PMC10284401

[B61] Wang J. Wang X. Yang K. Lu C. Fields B. Xu Y. . (2024). Phage selection drives resistance–virulence trade-offs in Ralstonia solanacearum plant-pathogenic bacterium irrespective of the growth temperature. Evol. Lett. 8, 253–266. doi: 10.1093/evlett/qrad056, PMID: 38525025 PMC10959482

[B62] Webby R. J. Webster R. G. (2001). Emergence of influenza A viruses. Philos. Trans. R. Soc. B 356, 1817–1828. doi: 10.1098/rstb.2001.0997, PMID: 11779380 PMC1088557

[B63] Wendling C. C. Lange J. Liesegang H. Sieber M. Pöhlein A. Bunk B. . (2022). Higher phage virulence accelerates the evolution of host resistance. Proc. R. Soc. B 289, 20221070. doi: 10.1098/rspb.2022.1070, PMID: 36196537 PMC9532999

[B64] Woolhouse M. E. J. Haydon D. T. Antia R. (2005). Emerging pathogens: The epidemiology and evolution of species jumps. Trends Ecol. Evol. 20, 238–244. doi: 10.1016/j.tree.2005.02.009, PMID: 16701375 PMC7119200

