# Supplementary material for: Hard to jump: host shifts appear unlikely in a T4-like phage evolved in the lab
Source: Front Cell Infect Microbiol. 2025 Nov 25;15:1597805. doi: 10.3389/fcimb.2025.1597805 (PMC12685909; doi:10.3389/fcimb.2025.1597805)
Supplement: Supplementary file 1 [file SupplementaryFile1.docx]

**Supplementary Information (S1)**

The phylogeny shown in Fig.1 is derived from a modified version of the alignment in Allen et al. (2017). For this alignment the Enterobase ([http://enterobase.warwick.ac.uk](http://enterobase.warwick.ac.uk/)) cgMLST pipeline was used to obtain the cgMLST profile of the 94 *E. coli* isolates. There were 1424 loci that had sequences for all isolates, these were aligned separately and concatenated (in order of genome location) into a 1.1 Mbp alignment. For this work the existing alignment was reduced to the strains included in this study (without re-alignment) and was re-run through RAxML (Stamatakis, 2014) under default parameters using the CIPRES (Cyber Infrastructure for Phylogenetic RESearch) portal to generate an unrooted tree. The tree was rooted manually according to the broad topology in Allen et al (2017).

Stamatakis A. 2014. RAxML version 8: a tool for phylogenetic analysis and post-analysis of large phylogenies. Bioinformatics 30:1312–1313.


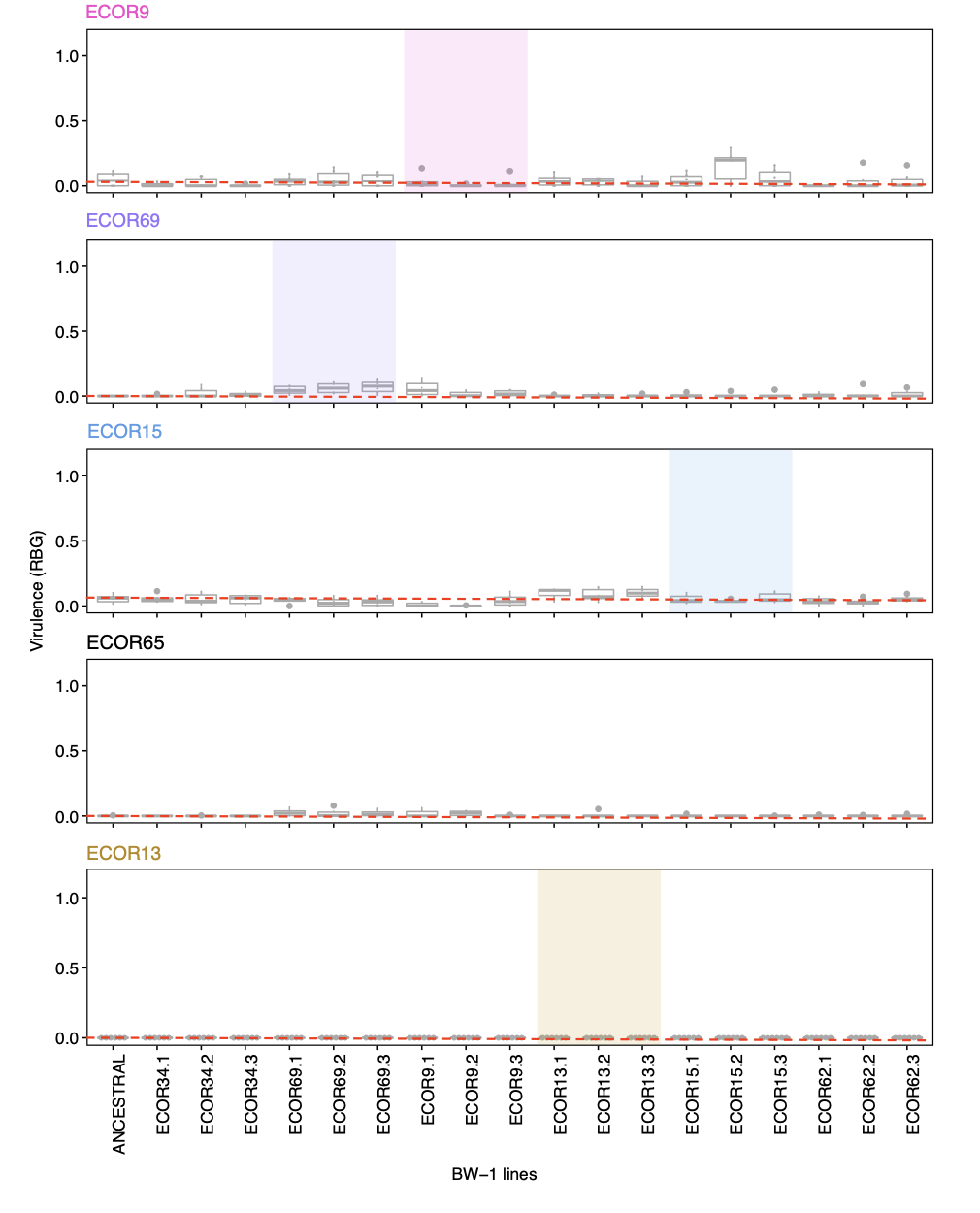


**Supplementary Figure 2 (S2).** No gains in virulence (RBG) of evolved phage lines towards non-permissive hosts. Each panel shows a different host strain, and the colored boxes show phage populations that evolved with that host strain. The dashed line represents the average virulence of the ancestral phage. Note that phage populations evolved with ECOR65 went extinct; therefore, there is no colored box in that panel.

**Supplementary Figure 3 (S3).** Virulence (RBG) of phage lines towards semi-permissive hosts. Each panel shows a different host strain, and the colored boxes show phage populations that evolved with that host strain. Dashed line represents average virulence of the ancestral phage. Within each host, asterisks denote significant differences in virulence tested as described in the main text. Note that phage populations evolved with ECOR36 went extinct and therefore there is no colored box in that panel.

**Supplementary Figure 4 (S4).** Virulence (RBG) of phage lines against the permissive host *E. coli* K12-MG1655. Dashed line represents average virulence of the ancestral phage. Asterisks denote significant differences in virulence tested as described in the main text.

**In excel file:**

Supplementary Table S1. Genome annotation of Escherichia phage BW-1 (Excel File)

Supplementary Table S2. Genomic differences between ancestral Escherichia phage BW-1 and the first derived Escherichia phage BW-1

Supplementary Table S3. Genomic differences between ancestral Escherichia phage BW-1 and the second derived Escherichia phage BW-1

Supplementary Table S4. Genomic differences between ancestral Escherichia phage BW-1 and the third derived Escherichia phage BW-1
